# IgG4-related kidney disease: the effects of a Rituximab-based immunosuppressive therapy

**DOI:** 10.18632/oncotarget.25095

**Published:** 2018-04-20

**Authors:** Giacomo Quattrocchio, Antonella Barreca, Andrea Demarchi, Laura Solfietti, Giulietta Beltrame, Roberta Fenoglio, Michela Ferro, Paola Mesiano, Stefano Murgia, Giulio Del Vecchio, Carlo Massara, Cristiana Rollino, Dario Roccatello

**Affiliations:** ^1^ Nephrology and Dialysis Unit, San Giovanni Bosco Hospital, and University of Turin, Italy; ^2^ Division of Pathology, Department of Medical Sciences, University of Turin, Turin, Italy; ^3^ Surgical Pathology Unit, San Giovanni Bosco Hospital, Turin, Italy; ^4^ Center of Research of Immunopathology and Rare Diseases (CMID), San Giovanni Bosco Hospital, and University of Turin, Turin, Italy

**Keywords:** IgG4-related disease, IgG4-related kidney disease, tubulointerstitial nephritis, retroperitoneal fibrosis, Rituximab

## Abstract

IgG4-related disease (IgG4-RD) is a recently recognized disorder, characterized by elevated serum IgG4 concentrations, dense tissue infiltration of IgG4-positive plasma cells and storiform fibrosis. Treatment is usually based on steroids, however, relapses and long-term adverse effects are frequent. We prospectively studied 5 consecutive patients with histologically-proven IgG4-RD and renal involvement, treated with an extended Rituximab protocol combined with steroids. Two doses of intravenous cyclophosphamide were added in 4 patients.

Five patients with IgG-RD were investigated: three had tubulointerstitial nephritis (TIN), while two had retroperitoneal fibrosis (RPF). In the patients with TIN, renal biospy was repeated after 1 year.

In the patients with TIN, estimated glomerular filtration rate (eGFR) at 12 months increased from 9 to 24 ml/min per 1.73 m^2^; IgG/IgG4 decreased from 3,236/665 to 706/51 mg/dl; C3/C4 increased from 49/6 to 99/27 mg/dl; CD20^+^ B-cells decreased from 8.7% to 0.5%; Regulatory T-cells decreased from 7.2% to 2.5%. These functional and immunologic changes persisted at 24 months and in two patients at 36 months. A repeat renal biopsy in the patients with TIN showed a dramatic decrease in interstitial plasma cell infiltrate with normalization of IgG4/IgG positive plasma cells. The patients with RPF showed a huge regression of retroperitoneal tissue.

In this sample of patients with aggressive IgG4-RD and renal involvement, treatment aimed at depleting B cells and decreasing antibody and cytokine production was associated with a substantial, persistent increase in eGFR, and a definite improvement in immunologic, radiologic and histological parameters.

## INTRODUCTION

IgG4-related disease (IgG4-RD) is a recently recognized, frequently multi-organ disorder characterized by tumefactive lesions or organ enlargement, elevated serum IgG4 concentrations, and peculiar histological features including dense lymphoplasmacytic infiltrate enriched with IgG4^+^ plasma cells, a storiform pattern of fibrosis, and obliterative phlebitis [1–2].

It predominantly affects middle-aged or elderly men, usually with a mild or subacute clinical presentation and it may mimic various malignant, infectious and inflammatory disorders [3].

Laboratory investigations show some very frequent, albeit non specific, abnormalities [4–6] including hypergammaglobulinemia (80–90% of cases), elevated (>135 mg/dl) serum IgG4 levels (50–70%), elevated serum IgE levels (60–70%), C3 and/or C4 hypocomplementemia (50–70%), peripheral eosinophilia (35–50%), antinuclear antibodies (30%) and rheumatoid factor (20–30%).

Cellular immunity, and particularly T-cells are implicated in disease pathogenesis. In fact, CD4^+^ T cells are abundant within IgG4-RD lesions, and tissue T cell cytokines (IL-4, IL-10 and TGF-β) are upregulated [7]. A polarized type 1 T helper or type 2 T helper cell population might act as a critical mediator of fibrosis by secreting cytokines such as IFN-γ and TGF-β which recruit and activate fibroblasts. A separate T follicular helper cell population producing IL-4 and IL-10 might promote class-switching of IgG antibodies to IgG4 and differentiation of B cells into plasma cells [2, 8, 9]. Lastly, regulatory T-cells likely play a central role in IgG4 and TGF-β production in the interstitium, thus promoting interstitial fibrosis [10].

The kidney is involved in approximately 15% of patients [4–6, 8]. Renal involvement, termed IgG4-related kidney disease (IgG4-RKD), may include a wide range of manifestations such as tubulointerstitial nephritis (TIN), membranous glomerulonephropathy (MGN), pyelitis, and hydronephrosis due to retroperitoneal fibrosis (RPF) [8, 11, 12]. Compared to general IgG-RD patients, IgG4-RKD patients (especially those with TIN) more frequently show hypocomplementemia and elevated serum IgG4 levels [5, 11–14].

IgG4-related TIN patients may present progressive renal failure, mild to moderate proteinuria, leukocyturia, and mild haematuria [8, 12, 15–17]. IgG4-related RPF patients may show only mild creatinine elevation or even normal renal function with normal urinalysis [12, 18].

Contrast-enhanced computerized tomography (CT), magnetic resonance imaging (MRI) and ^18^F-fluorodeoxyglucose (FDG) positron emission tomography/computed tomography (PET/CT) play an important diagnostic role in IgG4-RKD, in particular in patients with RPF, and are useful tools for monitoring therapeutic response and guiding interventional treatment [5, 12, 19].

Glucocorticoids are the first line of therapy for both IgG4-RD [1, 3, 9, 20] and IgG4-RKD [8, 15–17, 21], but disease relapse after tapering or discontinuing treatment is very high, and long-term use of glucocorticoids is associated with various adverse events [8, 9, 21]. Rituximab (RTX) has rarely been used as induction therapy but is frequently used as second-line therapy, especially in refractory cases [22–25].

We previously reported the favourable outcome of a cohort of patients with severe SLE treated with intensive B cell depletion therapy (IBCDT) consisting of a combination of 4 plus 2 infusions of RTX, cyclophosphamide and methylprednisolone pulses [26]. This approach made administering immunosuppressive maintenance therapy to avoid disease relapse unnecessary [27]. Results were confirmed in a larger series of patients with lupus nephritis followed-up for a mean of 44.5 months [28].

In the present study the effectiveness of IBCDT on clinical, biochemical, radiological and histologic parameters has been evaluated in patients with severe IgG4-related TIN and IgG4-related RPF.

## RESULTS

### Clinical and laboratory features of IgG4-RKD patients

Table 1 shows the baseline and follow-up data of all our patients. All the patients were followed-up for 24 months. IBCDT resulted in substantial renal functional improvement in all patients with TIN (#1, #2 and #3), who showed an increase in estimated glomerular filtration rate (eGFR) at 12 months from 9 (range, 8–11) to 24 (range, 14–36) ml/min per 1.73 m^2^. This improvement persisted at 24 months (26 ml/min per 1.73 m^2^, range, 19–37), and at 36 months in patients #1 and #2 (39 and 27 ml/min per 1.73 m^2^, respectively). eGFR values also improved at 12, 24 and 36 months in patient #4 (from 48 to 74 ml/min per 1.73 m^2^ ), while in patient #5, who had RPF and bilateral ureteral stents, values remained stable six months after stent removal (from 44 to 49 ml/min per 1.73 m^2^).

**Table 1:** Clinical, histologic, and laboratory features of patients

	*Patient* 1	*Patient* 2	*Patient* 3	*Patient* 4	*Patient* 5
Age (yr)	74	70	82	54	73
Sex	Male	Male	Male	Male	Female
IgG4-RKD	TIN	TIN	TIN	RPF	RPF
Baseline eGFR (ml/min per 1.73 m^2^)	11	8	8	48	63
12-month eGFR (ml/min per 1.73 m^2^)	36	23	14	75	44
24-month eGFR (ml/min per 1.73 m^2^)	37	22	19	75	49
36-month eGFR (ml/min per 1.73 m^2^)	39	27		74	
Baseline sIgG (mg/dl)	2,195	5,201	2,312	1,034	1,387
12-month sIgG (mg/dl)	721	826	571	839	372
24-month sIgG (mg/dl)	1,026	821	695	618	739
36-month sIgG (mg/dl)	1,421	898		652	
Baseline sIgG4 (mg/dl)	354	1,390	253	136	215
12-month sIgG4 (mg/dl)	39	80	36	70	N.A.
24-month sIgG4 (mg/dl)	215	37	17	42	19
36-month sIgG4 (mg/dl)	299	35		41	
Baseline sC3/C4 (mg/dl)	51/10	47/1	50/8	141/32	138/28
12-month sC3/C4 (mg/dl)	108/35	102/20	89/28	144/35	N.A.
24-month sC3/C4 (mg/dl)	130/56	120/25	90/30	117/29	123/30
36-month sC3/C4 (mg/dl)	95/27	100/18		136/31	
Baseline CD20^+^ (%)	10	3.1	13	6.6	10.8
12-month CD20^+^ (%)	1.2	0.09	0.0	5	0.0
24-month CD20^+^ (%)	1.2	0.08	4.4	0.0	5.6
36-month CD20^+^ (%)	1.4	0.16		0.1	
Baseline Tregs (%)	9.0	5.4	N.A.	5.0	1.9
12-month Tregs (%)	3.0	2.5	2.2	5.5	1.0
24-month Tregs (%)	3.8	4.9	6.1	2.6	5.0
36-month Tregs (%)	4.9	5.5		0.9	

Abbreviations: *yr*, year; *IgG4-RKD*, IgG4-related kidney disease; *TIN*, Tubulointerstitial nephritis; *RPF*, retroperitoneal fibrosis; *eGFR*, estimated glomerular filtration rate; *sIgG*, serum IgG; *Tregs*, regulatory T cells; *N.A.*, not available.

All immunologic parameters showed a definite improvement at 1 year in TIN patients (#1, #2 and #3). In particular, total serum IgG decreased from 3,236 (range, 2,195–5,201) to 706 (range, 571–826) mg/dl; likewise, IgG4 subclass decreased from 665 (range, 253–1,390) to 51 (range, 36–80) mg/dl. Complement mean level increased to normal: C3 went from 49 (range, 47–51) to 99 (range, 89–108) mg/dl; C4 rose from 6 (range, 1–10) to 27 (range, 20–35) mg/dl.

CD20^+^ B-cells decreased from the pre-treatment mean value of 8.7% (range, 3.1–13) to 0% seven days after the 4th infusion (data not shown), and showed a mild increase to 0.5% (range, 0.09–1.2) after 12–15 months.

Circulating regulatory T-cells (reference values, 0.5–5%) decreased from 7.2% (range, 5.4–9) to 2.5% (range, 2.2–3.0) at one year.

The overall changes in immunological parameters persisted at 24 months and in patients #1 and #2 even at 36 months. Interestingly, patient #3, who had not received cyclophosphamide due to a past history of colon cancer, showed a definite increase in CD20^+^ B-cells (from 0 to 4.4%) and Treg cells (from 2.2 to 6.1%) at 24 months.

In the two patients with RPF total IgG, IgG4, CD20^+^ B-cells and Treg cells decreased as expected due to the immunosuppressive treatment, while C3 and C4 levels, that were already normal, remained unchanged.

### Histologic features of IgG4-RKD patients

Patients #1, #2 and #3 underwent a second renal biopsy 12 months after the first procedure.

**Figure 1:**
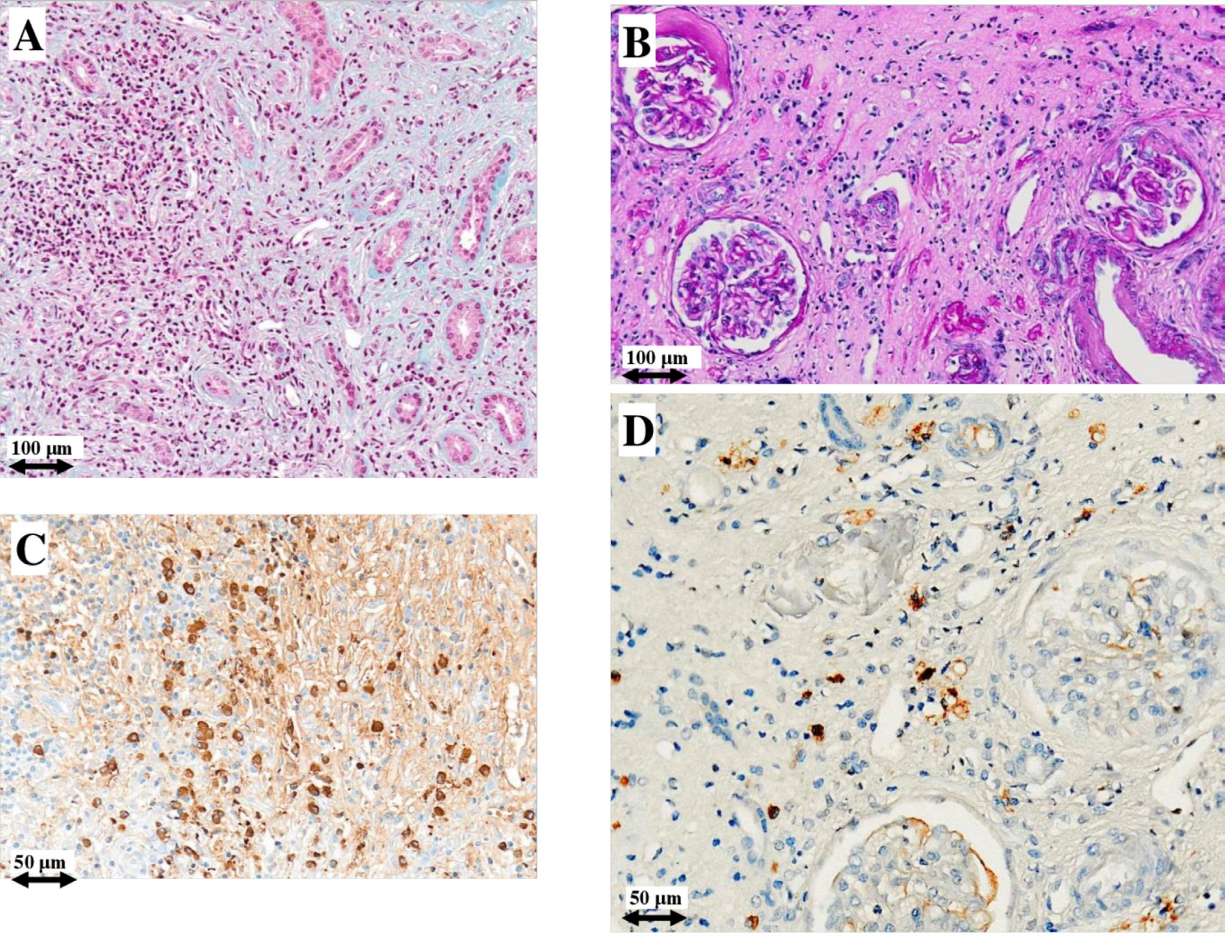
Light microscopy and immunohistochemistry findings in patient #1 Plasma cell infiltrate before (**A**) and after (**B**) intensive B cell depletion therapy (IBCDT). IgG^+^ plasma cells before (**C**) and after (**D**) IBCDT. (A) Masson's trichrome (original magnification ×100); (B) Periodic acid-Schiff solution (PAS) (original magnification ×100); (C, D) Immunohistochemical staining (original magnification ×200).

**Figure 2:**
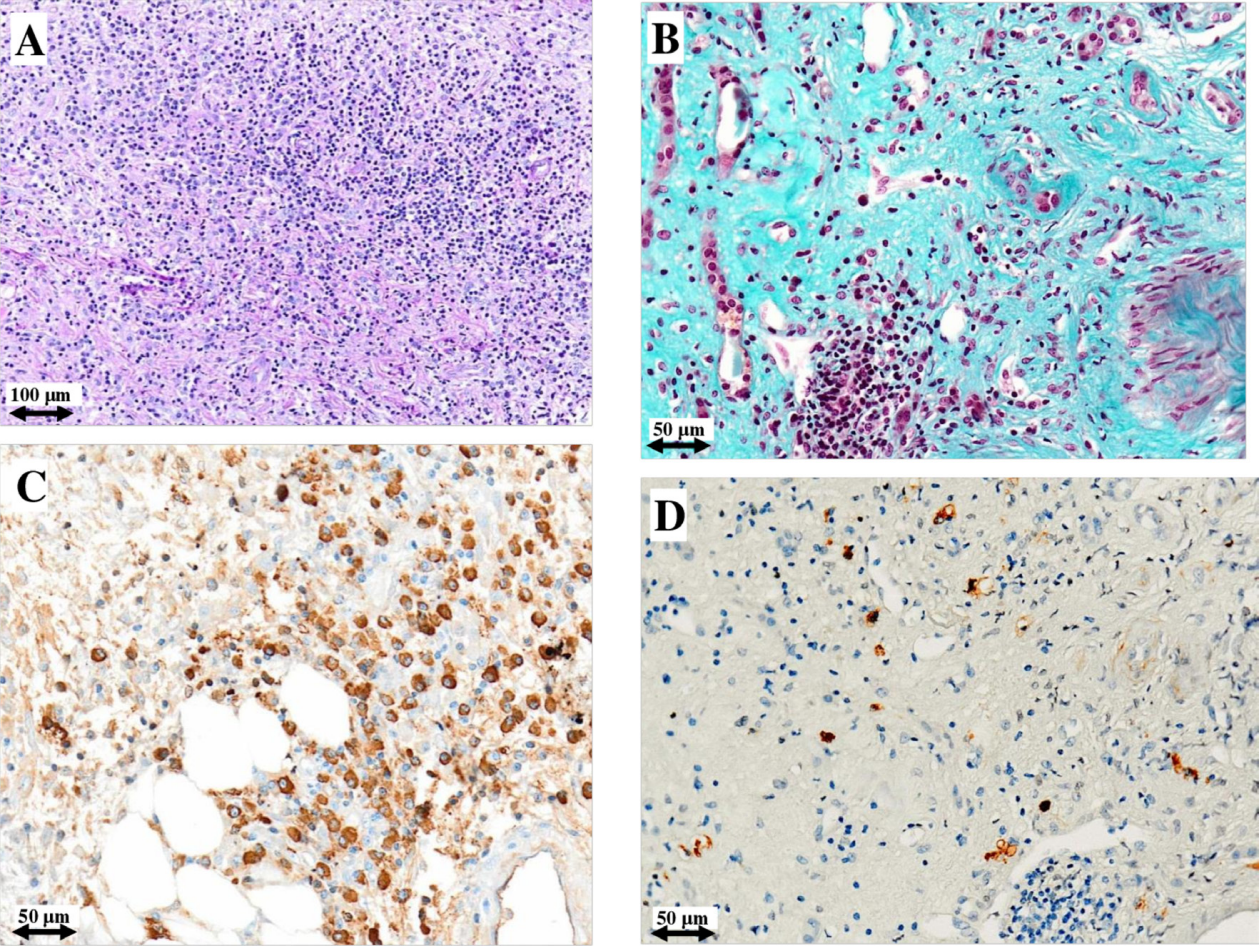
Light microscopy and immunohistochemistry findings in patient #2 Plasma cell infiltrate and storiform fibrosis before (**A**) and after (**B**) intensive B cell depletion therapy (IBCDT). IgG^+^ plasma cells before (**C**) and after (**D**) IBCDT. (A) Periodic acid-Schiff solution (PAS) (original magnification ×100); (B) Masson's trichrome (original magnification ×200); (C, D) Immunohistochemical staining (original magnification ×200).

Figures 1 and 2 depict the histological picture at diagnosis and one year later as shown by the serial biopsies performed on patients #1 and #2, respectively: light microscopy study revealed a remarkable reduction of interstitial plasma cell infiltrates. Furthermore, immunohistochemistry showed normalization of IgG4/IgG positive plasma cells (from 40% to 4% and from 60% to 2%, respectively). The repeat renal biopsy of patient #3 showed the same histological improvement.

In particular, the first biopsy in patient #1showed a more densely cellular inflammatory lesion with expansile storiform interstitial fibrosis involving about 80% of the examined material; in the second biopsy there was a marked reduction of storiform interstitial fibrosis that occupied only about 5% of the evaluated material, with an associated evident decrease of the inflammatory component. Furthermore, immunofluorescence of the first biopsy demonstrated the presence of granular immune deposits (IgG, IgM and C3) in the tubular basement membrane which became negative in the second one. In patient #2 the amount of interstitial fibrosis remained substantially unchanged between the two biopsies, conversely there were only scattered plasma cells with a normal IgG4/IgG ratio in the second one. In patient #3 there was a mild reduction of interstitial fibrosis between the two biopsies, but the second one showed only focal aspects with a storiform pattern and occasional plasma cells.

**Figure 3:**
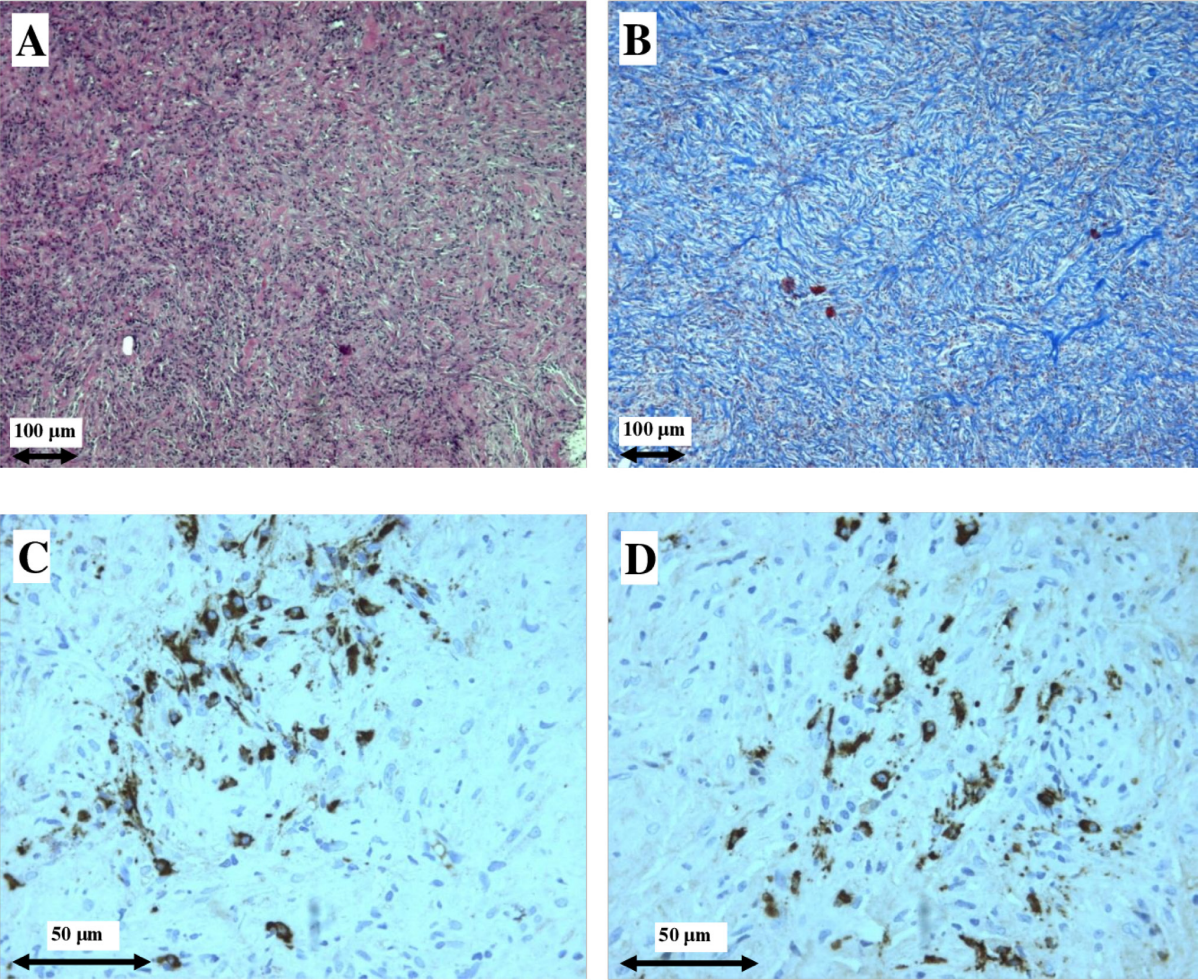
Light microscopy and immunohistochemistry findings in patient #4 Dense plasma cell infiltrate (**A**) and storiform fibrosis (**B**) in surgical periaortic tissue biopsy, typical of IgG4-related retroperitoneal fibrosis. IgG^+^ plasma cells (**C**), and IgG4^+^ plasma cells (**D**). (A) hematoxylin and eosin (original magnification ×100); (B) Masson's trichrome (original magnification ×100); (C, D) Immunohistochemical staining (original magnification ×400).

Figure 3 illustrates light microscopy and immunohistochemistry findings of the surgical periaortic tissue biopsy performed on patient #4 which shows a dense lymphoplasmacytic infiltrate, the characteristic storiform pattern of fibrosis and >30 IgG4^+^ plasma cells/HPF.

### Radiologic features of IgG4-RKD patients

**Figure 4:**
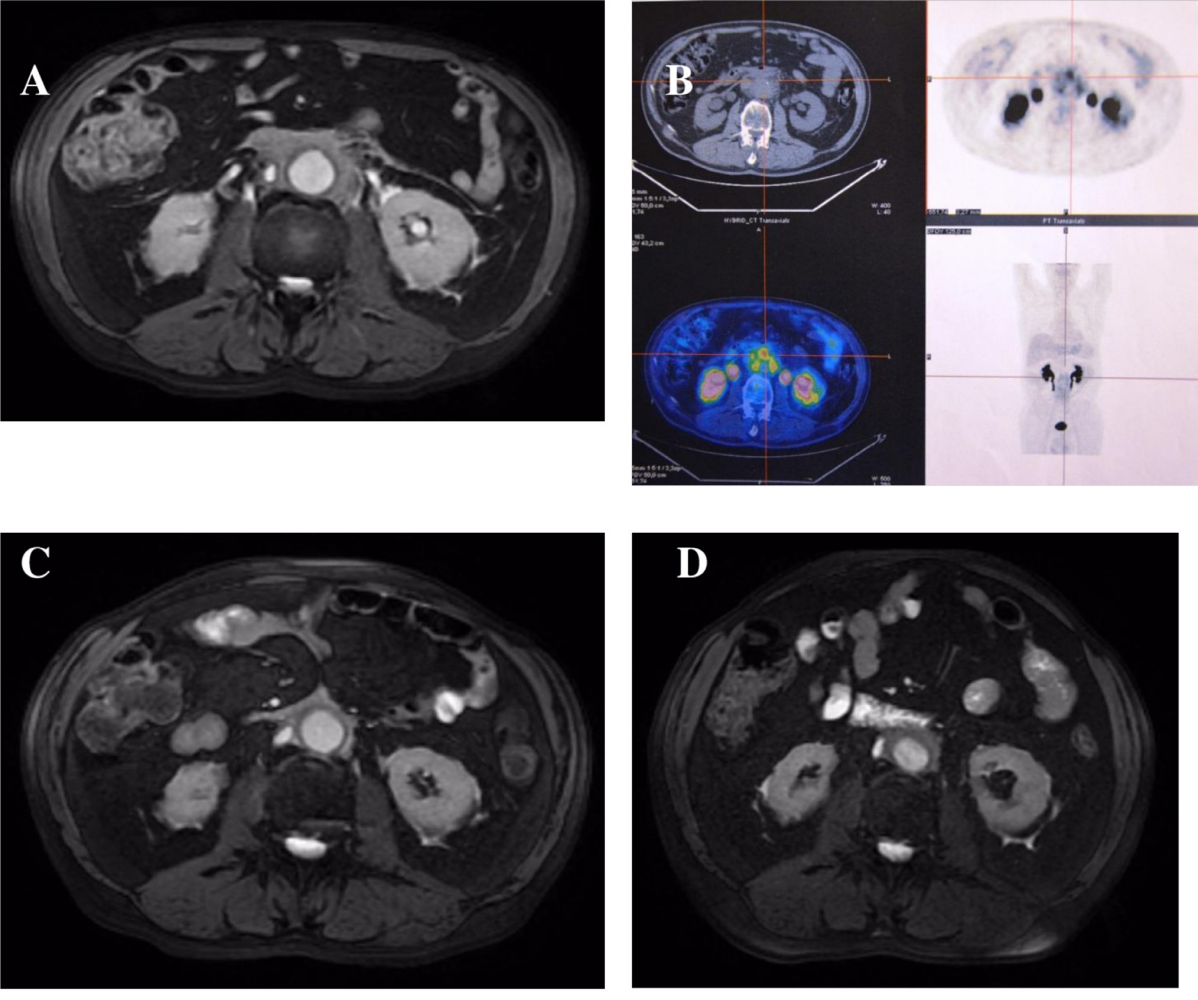
Radiologic features of patient #4 Magnetic resonance and PET/CT imagings before (**A, B**), 5 months (**C**), and 16 months (**D**) after treatment: (A and B) abnormal periaortic soft tissue, axial diameter 17 mm, showing high metabolic activity; (C) 60% reduction of tissue axial thickness; (D) 80% increase of periaortic mass axial diameter.

Figure 4 shows the evolution of retroperitoneal, periaortic tissue in patient #4: a 60% reduction in thickness was evident at 5 months, but a new increase in the fibro-inflammatory mass due to disease reactivation was seen at 16 months. Interestingly, none of the immunologic parameters (total IgG, IgG4, C3/C4, CD20^+^ B-cells, Treg cells) showed any changes that might suggest this disease relapse. The patient was therefore treated with oral steroids which were gradually tapered during the following 3 months, and with RTX 375 mg/m^2^ every 4 months, showing again a gradual reduction of retroperitoneal tissue in MRI and PET/CT studies.

**Figure 5:**
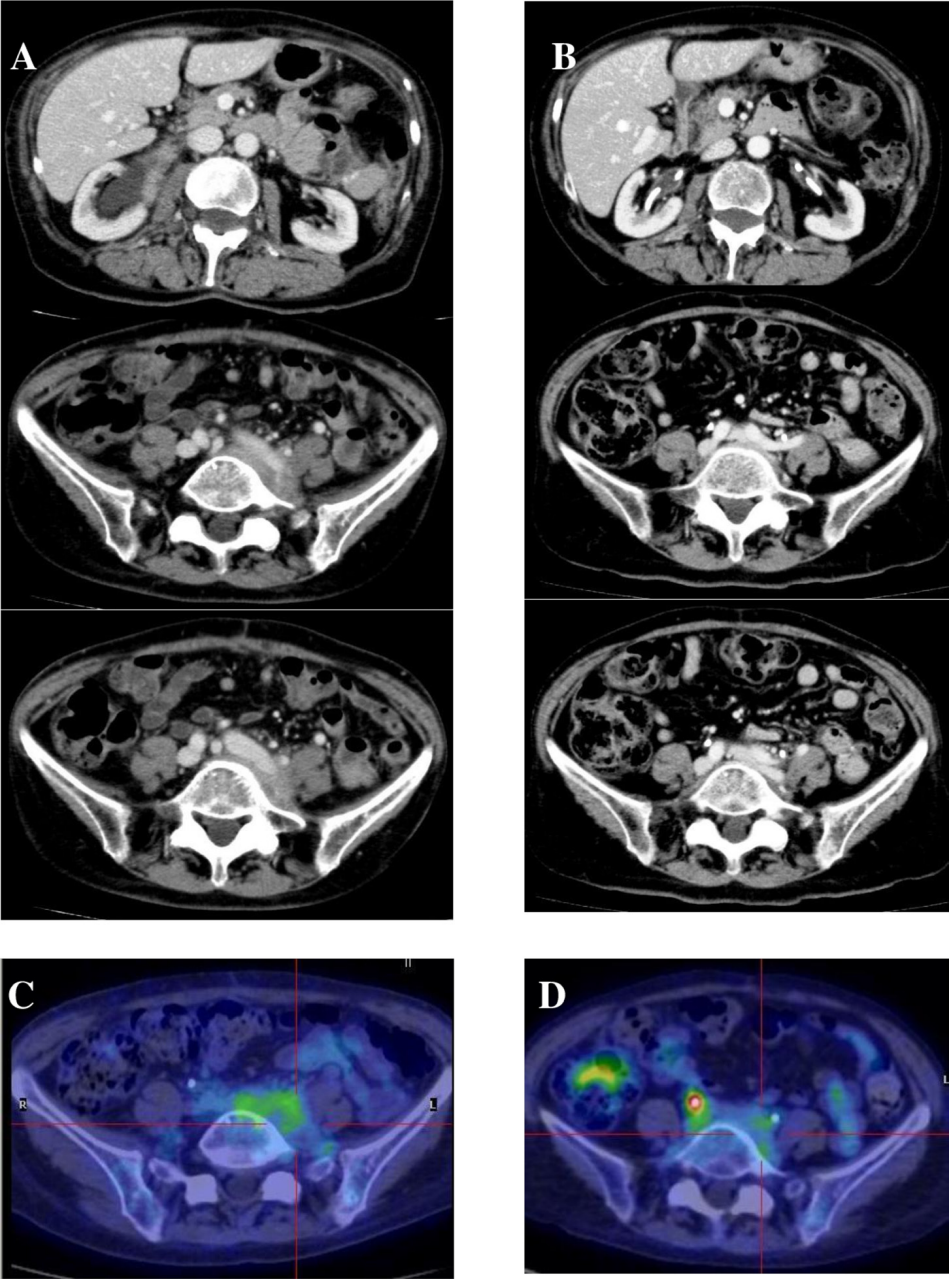
Radiologic features of patient #5 CT scan and PET/CT imagings before starting therapy (**A**, **C**) and at 6 months (**B, D**): (A) retroperitoneal and perivascular fibroinflammatory tissue, with (C) high metabolic activity; (B) complete disappearance of abnormal tissue, with (D) persistence of residual minimal pelvic metabolic activity.

Figure 5 shows radiologic imaging performed on patient #5 before starting the IBCDT protocol and at six months: complete disappearance of retroperitoneal and perivascular fibroinflammatory tissue on CT scan is evident, with persistent residual minimal pelvic metabolic activity on PET/CT study. After 14 months, ureteral stents were removed without the reappearance of hydronephrosis in the subsequent MRI study.

### Treatment-related complications of IgG4-RKD patients

Patients #1 and #2 developed steroid-induced diabetes mellitus requiring transient insulin therapy that was eventually discontinued in conjunction with prednisone discontinuation.

Patient #4 developed mild, acute bronchopneumonia during the course of immunosuppressive treatment, which was rapidly and completely controlled by oral antibiotics.

## DISCUSSION

Glucocorticoids represent the first line of therapy for both IgG4-RD (1, 2, 9, 20) and IgG4-RKD (8, 15–17, 21).

A starting prednisolone dose of 0.6–1.0 mg/kg/daily, gradually tapered after 2–4 weeks depending on the clinical response, is the usual approach for IgG4-RD patients with organ failure, in particular in cases of type 1 autoimmune pancreatitis, and clinical and laboratory improvements are frequently rapid, depending on the affected organs and the degree of fibrosis [1, 2, 9]. Nevertheless, although the initial response is often good, disease relapse after tapering or discontinuing steroids is very high [9]. Furthermore, steroid therapy exposes patients to several possible adverse side effects, so immunosuppressants such as azathioprine, mycophenolate mofetil, methotrexate and cyclosporine have been used as glucocorticoid-sparing agents, or in patients showing incomplete response. However, their efficacy has not been demonstrated [2, 9].

RTX has been shown to be effective in improving clinical and serologic features of IgG4-RD patients with active inflammation [20–23], even without concomitant steroid therapy [24]. Treatment with RTX can decrease serum IgG4, with serum IgG4 concentrations remaining low (and disease quiescent) even after B cell reconstitution [23].

Steroids are effective for most patients with IgG4-related TIN [13, 16, 21] and for some patients with IgG4-related RPF [18]. However, recovery of renal function may be incomplete. Furthermore, relapse of renal dysfunction following steroid tapering or discontinuation is frequently observed, and irreversible renal failure may occur, especially in patients with advanced renal damage [8, 17, 21]. Compared to glucocorticoids, RTX might yield a more effective and long-lasting response in IgG4-RKD patients. This is a relatively unexplored field because, apart from preliminary data from our group [12] and a recent report on IgG4-related aggressive TIN [30], RTX has not yet been used in the specific population of IgG4-RKD patients.

An international panel of experts very recently developed some recommendations for the management of IgG4-RD, confirming glucocorticoids as first-line agents for remission induction in all patients with active, untreated disease (94% agreement). Conversely, there was lower agreement (46%) on the use of steroid-sparing agents, including RTX [31], possibly due to the insufficient difference between results obtained with steroids compared to RTX given alone.

In the present case series, we treated 1 patient who had IgG4-related TIN with a combination therapy of steroids and RTX, and 4 patients (2 with IgG4-related TIN and 2 with IgG4-related RPF) with the intensified immunosuppressive protocol adopted in our Department for severe SLE nephritis [26, 28]. In these 4 patients 2 pulses of intravenous cyclophosphamide were added to steroids and RTX because of rapidly progressive renal failure or severe histological activity or high metabolic activity at radiologic investigation.

The rationale for the combination therapy with small doses of cyclophosphamide over rituximab alone relies both on taking advantage of the synergic effects with RTX on CD20 positive cells and on expanding immunomodulation over a wider spectrum of B cells like plasmablasts also inhibiting their antigen presenting cell function. In our opinion this intensified treatment could be taken into consideration in patients with very aggressive clinical and histologic forms of IgG4-RKD and who may require more rapid and efficient induction therapy. Due to the small sample of patients additional studies are required to confirm this observation.

Actually, patients with IgG4-related TIN, characterized by extremely reduced eGFR at presentation showed a remarkable response both in renal function and in immunologic parameters. Despite the severity of the disease in our series, these effects still persisted without any further immunomodulatory or anti-inflammatory therapy 2 years later, which is in line with the results already observed in severe SLE patients with long term follow-up (44 months) [28]. Furthermore, the three repeat renal biopsies performed one year after presentation, i.e., 6–8 months after completely discontinuing therapy, showed only modest plasma cell infiltration, further supporting the long term effects of IBCDT. Of note, with regard to the interstitial fibrosis, the peculiar storiform pattern characterizing IgG-RD was less evident [12]. Interestingly, patient #3, who did not receive cyclophosphamide due to a past history of colon cancer showed an increase of CD20^+^ B-cells (from 0 to 4.4%) and Treg cells (from 2.2 to 6.1%) at 24 months; the less aggressive immunosuppression may have facilitated this increase, but whether these immunologic modifications might predict disease relapse remains to be confirmed.

Normalization of circulating regulatory T-cells at the end of therapy, together with the functional, immunological and histological improvement observed in our TIN patients support a role for these cells in the pathogenesis of IgG4-RD as proposed by Takeuchi *et al.* [7] and suggest their potential usefulness in the follow-up of patients.

With regard to IgG4-related RPF, the high recurrence rate often requires maintenance immunosuppressive therapy [32]. The clinical course of our patient with the longer follow-up confirms the need for continuous monitoring with MRI or PET/CT (performed at 6–12 month- intervals for at least 3 years) in order to identify disease reactivation.

The main limitations of our study are the small number of patients, the population heterogeneity and the limited follow-up. In particular, the three patients with TIN showed different immunologic parameters as compared to RPF patients including higher levels of serum IgG, IgG4, circulating T regulatory cells, and lower complement levels. Some of these features have been already described [8, 11, 13, 14], and could suggest slightly different, organ-specific, pathogenetic mechanisms, but the actual significance remains to be elucidated. Notably, severe renal involvement is quite exceptional in IgG4-RD which is per se a very rare disease. This is a typical condition that makes the possibility of a randomized controlled study highly unrealistic.

The main strength of this study is that it is the first case series study to include repeat renal biopsies. Re-examination of histology one year after diagnosis and therapy helps guide further therapeutic decisions, provides insight into the possible progression of the disease or, as in our cases, supports discontinuation of therapy.

## MATERIALS AND METHODS

### Patients and design

Between October, 2013 and October, 2015 five consecutive patients (4 men and 1 woman) were found to have IgG4-related kidney disease in our Unit. Three patients had histologically-proven IgG4-related TIN, while two patients had histologically-proven IgG4-related RPF. Renal and retroperitoneal tissue specimens were examined by light microscopy and immunohistochemistry.

Diagnosis of IgG4-related TIN was based on Raissian's criteria [13], while histological evaluation of tissue specimens from the patients with RPF was performed according to the consensus statement proposed by Deshpande *et al.* [29].

A previous diagnosis of Sjogren-like syndrome had been made in patient #1, who was treated with small doses of oral prednisone. One year later, he was admitted to our Division for rapidly progressive renal failure. Renal biopsy showed an IgG4-related TIN.

Patient #2 had been mono-nephrectomized in another hospital due to a suspected neoplasm with a final histological diagnosis of xanthogranulomatous pyelonephritis 16 months before coming to our observation. He was admitted to our Division for rapidly progressive renal failure and underwent renal biopsy, which showed a typical IgG4-related TIN.

Patient #3 had undergone colon resection 20 years earlier due to a malignant tumour. He was admitted to our Division for acute renal failure overlapping chronic impairment. Renal biopsy showed an IgG4-related TIN.

Patient #4, who had been admitted for lower back pain and mild acute renal failure, showed bilateral hydroureteronephrosis due to retroperitoneal and periaortic abnormal tissue observed both at ultrasound and CT imaging. He underwent an exploratory laparotomy, and the tissue specimen showed features of IgG4-related RPF.

Patient #5 underwent radical hysteroannessiectomy 10 months earlier due to suspected uterine cancer with secondary, initially monolateral, hydroureteronephrosis. She required bilateral ureteral stent placement for recurrent hydronephrosis due to the presence of abnormal pelvic and periureteral tissue, characterized by high metabolic activity at the FDG-PET/CT. After admission to our Unit, re-examination of the surgical specimen confirmed the suspicion of histologically active IgG4-related RPF.

Estimated GFR using the CKD-EPI formula, blood count, IgG/IgG4, C3/C4, CD20^+^ cells and circulating Tregs were measured at baseline and during follow-up in all patients.

Contrast-enhanced CT or MRI and FDG-PET/CT were performed at baseline and during follow-up in both patients with RPF.

Percutaneous needle biopsy was repeated in all the patients with TIN one year after the initial histological diagnosis.

### Renal and periaortic tissue pathology

Renal samples were obtained by real-time ultrasound-guided percutaneous needle biopsy in patients #1, #2, and #3. Periaortic tissue was obtained by open biopsy of the mass lesion in patient #4 and by surgical hysteroannessiectomy in patient #5. Renal and periaortic tissue specimens were processed for light and immunofluorescence microscopy. Immunostaining was performed using anti-IgG and anti-IgG4 antibodies.

### Treatment protocol

Patients #1, #2, #4 and #5 were treated according to the IBCDT protocol previously described by our group in severe cases of SLE with nephritis [26]. They intravenously received 3 pulses of 15 mg/kg methylprednisolone followed by oral prednisone (0.8 mg/kg/day, gradually tapered until discontinuation in 4 months) *plus* 2 pulses of 500–750 mg cyclophosphamide (on days 1 and 15) *plus* 4 weekly Rituximab administrations (375 mg/m^2^). Two more doses of Rituximab were administered 1 and 2 months following the last weekly infusion.

In patient #3 cyclophosphamide was avoided due to his past history of colon cancer.

Patients #2 and #5 received isoniazid prophylaxis due to a positive QuantiFERON-TB Gold test result.

## CONCLUSIONS

IgG4-RKD may be a progressive disease that, if untreated, can lead to chronic renal failure and dialysis. Steroid treatment may be insufficient in the most severe forms of IgG4-TIN showing severe tubular atrophy and interstitial fibrosis as well as in cases of IgG4-RPF with widespread involvement. Furthermore its use is limited by several adverse effects, particularly in elderly patients.

In our small sample of patients with aggressive disease, IBCDT has proved to be effective and safe. These preliminary observations warrant future studies.
